# Pandemic fatigue and depressive symptoms among college students in the COVID-19 context: indirect effects through sense of control and intolerance of uncertainty

**DOI:** 10.1186/s40359-024-01521-2

**Published:** 2024-01-11

**Authors:** Qinglu Wu, Tian-Ming Zhang, Xue Wang, Yan Zhang

**Affiliations:** 1 Institute of Advanced Studies in Humanities and Social Sciences, Beijing Normal University, Zhuhai, China; 2 Department of Social Work, Shanghai University, Shanghai, China; 3 Business School, Beijing Normal University, Beijing, China; 4 School of Media and Communication, Shenzhen University, Rm 823, Zhiyi Building (L7), Ave No. 3688, Nanhai Road, Nanshan district, Shenzhen, 518000 China

**Keywords:** Pandemic fatigue, Sense of control, Intolerance of uncertainty, Depressive symptoms, COVID-19, Stress process model

## Abstract

Pandemic fatigue, the state of weariness, exhaustion, and demotivation to engage in protective behaviors during the coronavirus disease 2019 (COVID-19) pandemic, have been linked with depressive symptoms. However, the mechanism underlying this association remains unclear. Based on the stress process model, the present study examined the indirect effects of pandemic fatigue on depressive symptoms through the indirect roles of sense of control and intolerance of uncertainty. Data were collected from 1,162 Chinese undergraduate and postgraduate students (*M*_age_ = 21.61 years old, *SD* = 2.81, 64.29% women) through electronic questionnaires. The pandemic fatigue scale, the personal mastery scale, the short version of the Intolerance of Uncertainty Scale, and the Depression subscale of the Depression-Anxiety-Stress Scales were employed. Indirect effects were analyzed using structural equation modeling. The results revealed that pandemic fatigue related to COVID-19 was positively associated with depressive symptoms through (a) sense of control; (b) intolerance of uncertainty; and (c) a sequential pathway from sense of control to intolerance of uncertainty. The findings expand the application of the stress process model to the context of COVID-19 and deepen the understanding of pandemic fatigue—depressive symptoms link with considering the indirect roles of sense of control and intolerance of uncertainty. Psychological distress in the context of COVID-19 could be alleviated by the interventions on strengthening sense of control and tolerance of uncertainty and lessening the adverse effects of pandemic fatigue.

## Introduction

Since its onset in early 2020, the coronavirus disease 2019 (COVID-19) pandemic has profoundly affected the lives of billions of people worldwide. Because of the long-term effects of the COVID-19 pandemic, the World Health Organization (WHO) [1] has urged the public to remain cautious about pandemic fatigue, a phenomenon that has raised worldwide concerns [2]. Pandemic fatigue emerges because of prolonged exposure to COVID-19 information and related behavioral restrictions (e.g., social distancing and quarantine), which cause physical and mental exhaustion and negative attitudes toward adherence to protective behaviors [3]. Pandemic fatigue usually leads to adverse consequences, such as increased posttraumatic stress symptoms, information avoidance, and detrimental effects on sleep quality, job contentment, and personal resources (e.g., resilience) [3–5]. One consequence of pandemic fatigue is its effects on psychological distress (e.g., depressive symptoms, anxiety, and stress), especially depressive symptoms [6–8]. Although studies have revealed a positive association between pandemic fatigue and depressive symptoms, the underlying mechanism remains unexplored. Based on the stress process model [9, 10], the present study examined the indirect effects of pandemic fatigue on depressive symptoms through sense of control and intolerance of uncertainty.

Pandemic fatigue refers to the state of exhaustion and the demotivation to engage in protective behaviors that results from information overload and behavioral restrictions (i.e., information fatigue and behavior fatigue) during the COVID-19 pandemic [11]. Individuals may experience exhaustion or burnout from constant exposure to information about COVID-19 on social media and from behavioral restrictions (e.g., social distancing and quarantine). These negative experiences may cause exhaustion and decreased motivation or commitment to adhere to protective behaviors (e.g., mask wearing and social distancing). One far-reaching consequence of pandemic fatigue is poor mental health and increased psychological distress, especially depressive symptoms. Studies have revealed that pandemic fatigue is associated with depressive symptoms in different populations (e.g., teachers, college students, and community sample) [6–8]. Individuals who experience stress, exhaustion, and fatigue due to the COVID-19 pandemic are more likely to be depressed. The stress process model provides a theoretical framework to investigate the mechanism by which pandemic fatigue affects depressive symptoms. Such an investigation is crucial to develop related interventions to protect individuals’ mental health from pandemic fatigue in the context of COVID-19.

### Theoretical foundation

The stress process model describes how initial stressors (e.g., life difficulties, disasters, and chronic stressors) affect outcomes through the mediation of stress proliferation [10]. Individuals first encounter initial stressors, which may trigger secondary stressors as a direct consequence. The process of stress proliferation is the accumulation and expansion of stressors that result from the initial stressor. Secondary stressors can be negative experiences or compromised personal resources (e.g., personal and social), such as self-esteem or social support [9]. Both initial and secondary stressors can produce intense stress or strain. Negative experiences and compromised or damaged personal resources resulting from stressors may further undermine personal mental health. Studies employing the stress process model have revealed that adverse childhood experiences (initial stressors) may affect mental health and subjective well-being (outcomes) in adulthood through facilitating negative experiences (e.g., exposure to other forms of violence) or undermining personal resources (e.g., self-esteem and self-compassion) [12–14]. By incorporating this framework in the context of COVID-19, this study posits that a decreased sense of control and increased intolerance of uncertainty could be the consequences of pandemic fatigue. Pandemic fatigue occurs because of information overload and negative emotions (e.g., exhaustion) caused by pandemic restrictions, and this initial stressor may lead to stress proliferation involving compromised personal resources in the form of decreased sense of control and increased intolerance of uncertainty. These secondary stressors further contribute to negative outcomes, such as depressive symptoms.

### The potential indirect effects through sense of control and intolerance of uncertainty

Sense of control is an adaptive value and a motivational factor that refers to an individual’s belief that they can influence their outcomes through their own efforts [15]. Sense of control is a valuable personal resource that can help enhance psychological well-being (e.g., fulfillment and life satisfaction) and health-related behaviors (e.g., increased physical activity and reduced smartphone use) and alleviate psychological distress, especially depressive symptoms [16–18]. Individuals who maintain a sense of control generally feel empowered in their lives and are less likely to feel depressed or hopeless about the future [17]. Although having a sense of control can alleviate depressive symptoms, this personal resource can be compromised by the stressors resulting from the pandemic. Studies have asserted that fear of COVID-19 and work-related stress in the context of COVID-19 are negatively associated with individuals’ sense of control [16, 19, 20]. Individuals who perceive COVID-19 as a great threat experience a lack of competence during the pandemic (i.e., decreased sense of control and increased powerlessness), which may result in aggressive tendencies [19]. Pandemic fatigue is a stressor caused by information overload and restrictions related to the COVID-19 pandemic. This study hypothesizes that (a) pandemic fatigue is indirectly associated with depressive symptoms through sense of control (pandemic fatigue → sense of control → depressive symptoms).

Intolerance of uncertainty refers to the inability to withstand uncertain or ambiguous events or situations, regardless of the possibility of negative outcomes [21, 22]. The COVID-19 pandemic has caused many individuals to become intolerant of uncertainty, resulting in detrimental consequences such as psychological distress (e.g., depressive symptoms and anxiety) and decreased positivity and mindfulness [21, 23, 24]. The longitudinal association between intolerance of uncertainty and depressive symptoms has been identified [21]. Intolerance of uncertainty is associated with depressive symptoms through worry [25]. The effects of intolerance of uncertainty may be enhanced in the context of COVID-19. Individuals are confronted with multiple uncertainties during the COVID-19 pandemic, such as the length of the pandemic, the government restrictions, and the events that may occur in the future [26]. Individuals experience higher levels of intolerance of uncertainty as the pandemic continues and stress levels persist. Studies have revealed that stressors such as the fear of COVID-19 are associated with psychological distress and decreased positivity through intolerance of uncertainty [23, 27]. Pandemic fatigue, a stressor caused by the long-term effects of COVID-19, causes physical and emotional exhaustion [3]. Therefore, individuals with higher levels of pandemic fatigue may be less able to tolerate uncertainty and thus may become more depressed. This study hypothesizes that (b) pandemic fatigue is indirectly associated with depressive symptoms through intolerance of uncertainty (pandemic fatigue → intolerance of uncertainty → depressive symptoms).

### The relationship between sense of control and intolerance of uncertainty

Studies have examined the relationship between sense of control and intolerance of uncertainty. Those studies have revealed that sense of control predicts intolerance or avoidance of uncertainty [28, 29]. Sense of control was found to be associated with anxiety through intolerance of uncertainty, and it played a mediating role in the relationship between future perspective and intolerance of uncertainty among college students and adults. Individuals with a strong sense of control generally feel secure, have a positive attitude toward life, and confront challenges with an optimistic mindset. These individuals believe that life difficulties can be influenced and managed through their own efforts, and that they can minimize or avoid situational uncertainty [30]. Thus, they are more likely to possess active expectations for the future and tolerate temporal uncertainty. Because possessing a sense of control can reduce intolerance of uncertainty, we hypothesize that pandemic fatigue is indirectly associated with depressive symptoms through (c) a sequential pathway from sense of control to intolerance of uncertainty (pandemic fatigue → sense of control → intolerance of uncertainty → depressive symptoms).

### The present study

**Fig. 1:**
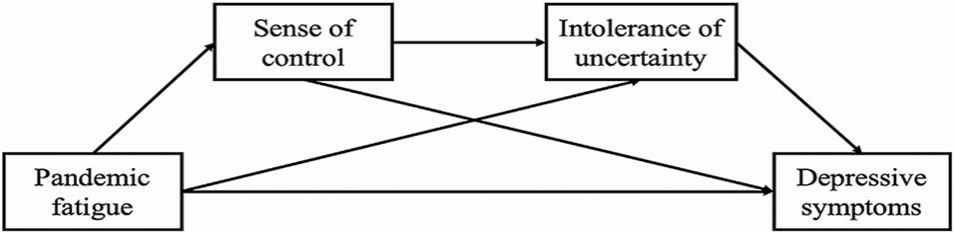
The hypothesized model

Studies have revealed an association between pandemic fatigue and depressive symptoms in the context of COVID-19 [6, 7], but the mechanism underlying this association remains underexplored. Investigating this association can provide valuable insights into the specific pathways in this association and can aid in the development of interventions or treatments for preventing the negative effects of pandemic fatigue. The present study fills this research gap by investigating how pandemic fatigue is associated with depressive symptoms based on the stress process model. This study is the first to examine the indirect roles of sense of control and intolerance of uncertainty in the association between pandemic fatigue and depressive symptoms. The initial stressor of pandemic fatigue may exert adverse effects on depressive symptoms by promoting the secondary stressors of compromised personal resources (i.e., sense of control) and negative experiences (i.e., intolerance of uncertainty). These findings contribute to the understanding of the mechanisms related to pandemic fatigue and broaden the application of the stress process model in the context of COVID-19. We hypothesize that pandemic fatigue is directly and indirectly associated with depressive symptoms through the following three potential indirect effects including (a) sense of control (pandemic fatigue → sense of control → depressive symptoms), (b) intolerance of uncertainty (pandemic fatigue → intolerance of uncertainty → depressive symptoms), and (c) a sequential pathway from sense of control to intolerance of uncertainty (pandemic fatigue → sense of control → intolerance of uncertainty → depressive symptoms). Figure 1 displays the hypothesized model in the present study.

## Methods

### Participants and procedure

This study employed data from a project on the effects of the COVID-19 pandemic (e.g., fatigue and public stigma) on mental health and well-being. Convenience sampling was employed. Participants in the project were Chinese college students who were 18 years or older. Recruiting information, which was distributed to students at Beijing Normal University, invited undergraduates and postgraduates to participate in an online survey in April 2022. Before data collection, the project was approved by the research ethics committee of the School of Social Development and Public Policy at Beijing Normal University. Participation in the project was voluntary, and participants were informed about the research purpose, procedures, confidentiality, and their right to withdraw from the project without consequences. Eligible participants received a small monetary reward (RMB 30, approximately US$4.5).

A total of 1,317 eligible participants completed the online survey. After excluding cases with duplicate responses (*n* = 37) and those that failed the attention check (*n* = 118), the final sample size was 1,162 (415 men, 747 women). The average age of the participants was 21.61 years (*SD* = 2.81). The percentages of undergraduates and postgraduates were 65.2% and 34.8%, respectively. The monthly household income was RMB19,811.49 (*SD* = 48,045.29, approximately US$2,968.90).

### Measures

Pandemic fatigue was measured using the pandemic fatigue scale [11]. This scale comprises 6 items assessed on a 7-point Likert scale ranging from 1 (*strongly disagree*) to 5 (*strongly agree*). Sample items include “I feel strained from following all the behavioral regulations and recommendations around COVID-19” and “I am losing my spirit to fight against COVID-19.” Higher mean scores indicate higher levels of pandemic fatigue. The Cronbach’s *α* for this scale was 0.91 in the present study.

Sense of control was assessed using the personal mastery scale [15]. This scale comprises 4 items measured on a 7-point Likert scale ranging from 1 (*strongly disagree*) to 7 (*strongly agree*). Example items include “Whether or not I am able to get what I want is in my own hands” and “What happens to me in the future mostly depends on me.” Higher mean scores indicate higher levels of sense of control. The Cronbach’s *α* for this scale was 0.80 in the present study.

Intolerance of uncertainty was measured using the short version of the Intolerance of Uncertainty Scale [22]. This scale was validated into Chinese version that comprises 11 items assessed on a 5-point Likert scale ranging from 1 (*not at all characteristic of me*) to 5 (*entirely characteristic of me*) [31]. Sample items include “When I am uncertain, I can not function well” and “Unforeseen events upset me greatly.” Higher mean scores indicate higher levels of intolerance of uncertainty. The Cronbach’s *α* for this scale was 0.93 in the present study.

Depressive symptoms were assessed using the Depression subscale of the Depression-Anxiety-Stress Scale [32]. Participants rated 7 items on a 4-point scale ranging from 1 (*did not apply to me at all*) to 4 (*applied to me very much*). Sample items include “I felt downhearted and blue” and “I felt that I had nothing to look forward to.” Higher mean scores indicate higher levels of depressive symptoms. The Cronbach’s *α* for this scale was 0.89 in the present study.

### Data analysis

This study implemented an initial descriptive analysis (e.g., mean, SD, internal consistency coefficients) and an indirect effects analysis by using SPSS (version 24) and Mplus (version 7.4), respectively. The indirect effects analysis was implemented using structural equation modeling. The model’s goodness of fit was assessed using the chi-square with its degree of freedom, the root mean square error of approximation (RMSEA), the comparative fit index (CFI), and the standardized root mean square residual (SRMR). An acceptable model fit was defined as RMSEA < 0.08, CFI > 0.90, and SRMR < 0.08 [33]. Resampling bias-corrected bootstrapping (5000 times) was employed to assess the sampling distribution of indirect effects. The significance of the indirect effects was evaluated using bias-corrected 95% confidence intervals (CIs). A CI of the indirect effect that does not include zero indicates a significant indirect effect. The analyses were controlled for age, sex, educational background, monthly household income, and whether the participants or people they knew had been infected with COVID-19.

## Results

The descriptive statistics and bivariate correlations of key variables are presented in Table 1. The results indicated positive associations between pandemic fatigue, intolerance of uncertainty, and depressive symptoms. Sense of control was negatively associated with pandemic fatigue, intolerance of uncertainty, and depressive symptoms. Variance inflation factors (VIFs) were employed to evaluate multicollinearity [34]. To calculate VIFs, we implemented regression analysis with depressive symptoms as the dependent variable and other key variables as independent variables. A VIF value more than or equal to 5 is generally regarded to indicate moderate multicollinearity. In the present study, all the VIFs of key variables were less than 5 (pandemic fatigue: 1.164, sense of control: 1.023, intolerance of uncertainty: 1.171); therefore, multicollinearity was not a concern in our model. In addition, Harman’s single-factor test was implemented to test for common method variance [35, 36]. Exploratory factor analysis was conducted using items of key variables to examine if a single general factor accounted for most of the covariance among the measures. The results revealed that four factors accounted for 62.65% of the total variance. The first unrotated factor accounted for 33.02% of the variance, indicating that common method bias did not substantially affect the model. Confirmatory factor analysis was implemented to validate the measurement model of the key variables. The results indicated that the measurement model adequately fit the data (*χ*^2^ = 1,105.284, *df* = 317, CFI = 0.958, SRMR = 0.055, RMSEA = 0.046, 90% CI [0.043, 0.049]).

**Table 1:** Descriptive statistics and correlations among the key variables

	Mean	SD	Range	1	2	3	4
1. Pandemic fatigue	3.18	1.48	1–7	—			
2. Sense of control	5.09	1.02	1–7	− 0.11^***^	—		
3. Intolerance of uncertainty	3.04	0.85	1–5	0.37^***^	− 0.13^***^	—	
4. Depressive symptoms	1.59	0.62	1–4	0.43^***^	− 0.21^***^	0.44^***^	—

*Note*: *p <.05, **p <.01, ***p <.001

**Fig. 2:**
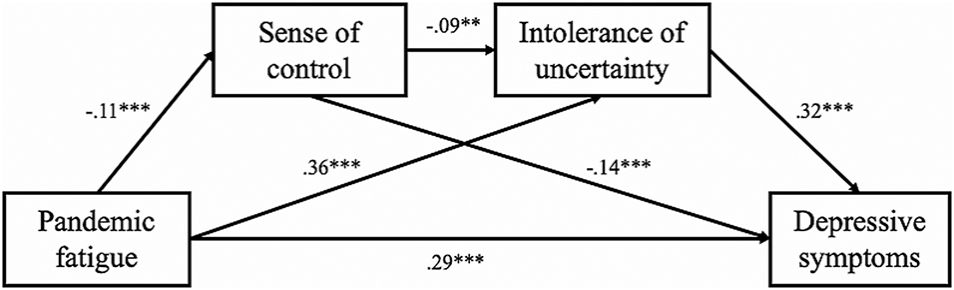
Paths and standardized path coefficients for the hypothesized model

The fit of the hypothesized model was acceptable (*χ*^2^ = 41.353, *df* = 10, *p* <.001, CFI = 0.951, SRMR = 0.025, RMSEA = 0.052, 90% CI [0.036, 0.069]). The results indicated that pandemic fatigue was positively and directly associated with depressive symptoms (*β* = 0.286, SE = 0.026, *p* <.001). Regarding indirect effects, pandemic fatigue was associated with depressive symptoms through sense of control (*β* = 0.016, SE = 0.005, 95% CI [0.007, 0.029]), intolerance of uncertainty (*β* = 0.113, SE = 0.012, 95% CI [0.090, 0.139]), and a sequential pathway from sense of control to intolerance of uncertainty (*β* = 0.003, SE = 0.001, 95% CI [0.001, 0.007]). The coefficients of the direct and indirect effects of pandemic fatigue on depressive symptoms are displayed in Table 2. The results revealed significant associations between depressive symptoms and the covariates of educational background (*β* = −0.101, SE = 0.036, *p* =.005), and whether the participants or people they know had been infected with COVID-19 (*β* = −0.071, SE = 0.025, *p* =.004). Independent variables accounted for 30.6% of the variance in depressive symptoms (*r*^2^ = 0.306, SE = 0.023, *p* <.001). The proportions of variance explained by direct effect and indirect effects including sense of control, intolerance of uncertainty, and a sequential pathway from sense of control to intolerance of uncertainty were 68.4%, 3.8%, 27%, and 0.7% respectively. The specific paths and standardized path coefficients for the hypothesized model are presented in Fig. 2.

**Table 2:** Direct and indirect effects of pandemic fatigue on depressive symptoms

Paths	Standardized parameter estimates	S.E.	Bias-corrected CI (95%)	
			Lower	Upper
Direct effect	0.286	0.026	0.227	0.343
Indirect effects				
PF → SC → DS	0.016	0.005	0.007	0.029
PF → IU → DS	0.113	0.012	0.090	0.139
PF → SC → IU → DS	0.003	0.001	0.001	0.007

*Note*: PF = pandemic fatigue, SC = sense of control, IU = intolerance of uncertainty, DS = depressive symptoms

## Discussion

The present study investigated how pandemic fatigue exerts its effect on depressive symptoms among Chinese college students. The results revealed that pandemic fatigue was directly and indirectly associated with depressive symptoms through (a) sense of control, (b) intolerance of uncertainty, and (c) a sequential pathway from sense of control to intolerance of uncertainty. Our findings support those of relevant studies asserting that the COVID-19 pandemic is generally associated with fatigue, stress, and impaired mental health among college students [3, 5, 16]. College students are vulnerable to the negative consequences of pandemic fatigue. College students who experience prolonged exposure to COVID-19-related information and behavioral restrictions are more likely to experience exhaustion and demotivation to practice protective behaviors [3]. Pandemic fatigue exerts far-reaching negative effects on mental health, especially with respect to depressive symptoms [8]. Thus, examining the underlying mechanism in the associations between pandemic fatigue and its outcomes yields valuable insights and enables the development of prevention strategies.

The results of the present study revealed a positive association between pandemic fatigue and depressive symptoms. This finding supports that the COVID-19 pandemic poses a considerable health-related challenge and implies the negative effects of prolonged exposure to COVID-19-related information and adherence to long-term preventive measures (e.g., social distancing and stay-at-home policies) on mental health [3, 37]. Although individuals may have been willing and able to follow preventive policies during the early stage of the pandemic, mental pressure and the unwillingness to adhere to restrictions tend to intensify as time passes. Similar to individuals who have experienced an illness [38], individuals who have endured a long-lasting pandemic may experience the lack of legitimation of the pandemic and ambiguity regarding the end of the pandemic. Consistent with the findings of relevant studies, the findings of this study indicate that pandemic fatigue reflects the exhaustion and helplessness experienced by individuals toward the pandemic and their demotivation to follow preventive measures, which could undermine their mental health and cause psychological distress, such as depressive symptoms [6, 7].

The results support our first and second hypotheses stating that pandemic fatigue is indirectly associated with depressive symptoms through (a) sense of control and (b) intolerance of uncertainty. Similar to previous findings indicating the negative effects of COVID-19-related stressors (e.g., fear and threat of COVID-19) on sense of control [16, 19, 23], sense of control and tolerance of uncertainty have been found vulnerable to the pandemic fatigue in the present study. When individuals become fatigued because of information overload and behavioral restrictions related to the COVID-19 pandemic, they may struggle to maintain their tolerance of uncertainty and sense of personal mastery over their life, which may lead to emotional distress and depression. These findings align with the stress process model [10]; information fatigue and behavioral fatigue are the initial stressors that induce feelings of exhaustion, and these negative experiences promote secondary stressors by weakening personal resources (i.e., sense of control) and enhancing risk factors (i.e., increased intolerance of uncertainty), which affect individuals’ well-being and mental health.

The study results also support our third hypothesis, which posits that pandemic fatigue is indirectly associated with depressive symptoms through (c) a sequential pathway from sense of control to intolerance of uncertainty. This result is consistent with previous findings on the relationship between sense of control and intolerance of uncertainty [28, 29], indicating that a lack of personal resources reduces an individual’s ability to tolerate uncertainty in life. When people have high strong sense of control, they feel optimistic about their circumstances and believe that they can resolve problems through personal efforts and the minimization of uncertainty [17, 30]. Thus, such individuals tend to maintain positive expectations about the future and can tolerate uncertainty. However, pandemic fatigue decreases individuals’ sense of control and confidence in managing life through personal efforts, creating unpredictable circumstances in the context of COVID-19. Compromised personal resources (i.e., sense of control) lead to a diminished ability to tolerate uncertainty (i.e., increased intolerance of uncertainty) and cause feelings of helplessness and depression.

Overall, our findings revealed that pandemic fatigue affects depressive symptoms among Chinese college students through three pathways, namely, sense of control, intolerance of uncertainty, and a sequential pathway from sense of control to intolerance of uncertainty. The findings align with the stress process model, which posits that an initial stressor causes negative consequences through the mediation of stress proliferation (i.e., causing the secondary stressors of negative experiences and compromised personal resources), and that both initial and secondary stressors can produce intense stress or strain [9, 10]. Pandemic fatigue, the initial stressor in the context of COVID-19, may lead to intolerance of uncertainty and a compromised sense of control, which promote psychological distress among college students. In addition, this study identified association between secondary stressors. Sense of control was associated with intolerance of uncertainty in the relationship between pandemic fatigue and depressive symptoms. College students who experience a decreased sense of control because of pandemic fatigue are less able to tolerate uncertainty. These findings provide insights into the mechanism by which pandemic fatigue affects mental health and broaden the application of the stress process model in the context of COVID-19.

### Limitations and implications

The present study has several limitations that must be addressed. First, this study employed a cross-sectional design to examine the mechanism underlying the associations between pandemic fatigue and depressive symptoms. Therefore, causal relationships between the key variables could not be examined. Future studies can employ a longitudinal design to analyze predictability over time. Second, the study used a sample from mainland China, where pandemic prevention measures were more frequently implemented during the data collection period. Whether our findings can be generalized to other countries in the context of COVID-19 requires additional investigation, especially for countries with less strict preventive measures. In addition, the local severity of the pandemic may affect mental health; therefore, data on the local severity of the pandemic should be collected and incorporated as a covariate in data analysis. Third, the study examined a sample of college students, which limits the generalizability of our findings to other populations. Because responses to the pandemic may vary by population and generation (e.g., perceived societal risk and preventive intention) [39], future studies can apply the hypothesized model to other populations, such as individuals who experience work stress due to the pandemic. Fourth, the investigation of factors contributing to the indirect effects of pandemic fatigue on depressive symptoms were limited to the individual level. Studies based on the spillover perspective have revealed that COVID-19-related stress could affect interpersonal factors (e.g., relationship satisfaction and parenting dynamics), which further contributes to personal distress and the development of depressive symptoms [40, 41]. Future studies can examine whether pandemic fatigue is associated with depressive symptoms through the interplay between interpersonal factors.

Although the present study has several limitations, the study findings have substantial theoretical and practical implications. The findings deepen our understanding of the indirect effects of pandemic fatigue on depressive symptoms by revealing the roles of sense of control and intolerance of uncertainty in this association. In addition, the findings broaden the applications of the stress process model [9, 10] in explaining the mechanism underlying the association between fatigue and psychological distress in the context of COVID-19. Pandemic fatigue functions as an initial stressor that triggers secondary stressors, including compromised personal resources (i.e., decreased sense of control) and negative experiences (i.e., increased intolerance of uncertainty), which further enhance psychological distress. Our findings provide practical insights into mental health care and interventions for depressive symptoms caused by COVID-19 pandemic fatigue. To combat the negative effects of pandemic fatigue on mental health, practitioners can develop interventions to strengthen individuals’ sense of control and tolerance of uncertainty (e.g., interventions related to personal mastery and cognitive bias modification program focused on intolerance of uncertainty) [42, 43], which may alleviate the psychological distress caused by high pandemic fatigue. In addition, this study recommends developing related campaigns and public education initiatives that focus on strategies aimed at mitigating the adverse effects of the pandemic (e.g., ensuring adequate supply of essential living materials and increasing access to medical services). Such measures can enable individuals to gain a greater sense of control in their life.

## Conclusions

The present study identified the indirect effects of pandemic fatigue on depressive symptoms through sense of control, intolerance of uncertainty, and a sequential pathway from sense of control to intolerance of uncertainty among Chinese college students. Our findings broaden the understanding of liking mechanism between stressor and its mental health outcome in the COVID-19 context with the support of stress process model. Initial stressor of pandemic fatigue may lead to mental health problem through proliferating into secondary stressors of compromised sense of control and deteriorated intolerance of uncertainty and their linkage. For the interventions aiming at reducing the negative effect of public stress resulted from COVID-19 pandemic on mental health, improving citizens’ sense of control and decreasing their intolerance of uncertainty could be potential working points.

## Data Availability

The datasets generated and/or analyzed during the current study are not publicly available due to ethical issues but are available from the corresponding author on reasonable request.

## Declarations

This manuscript is not under review elsewhere and the results have not been published previously or accepted for publication. This manuscript has been seen and approved by all authors.
